# Agreement in All-in-One Dataset between Diagnosis and Prescribed Medication for Common Cardiometabolic Diseases in the NDB-K7Ps

**DOI:** 10.3390/epidemiologia4040034

**Published:** 2023-10-02

**Authors:** Airi Sekine, Kei Nakajima

**Affiliations:** 1 Department of Food and Nutrition, Faculty of Human Sciences and Design, Japan Women’s University, Tokyo 112-8681, Japan; sekinea@fc.jwu.ac.jp; 2 Department of Endocrinology and Diabetes, Saitama Medical Center, Saitama Medical University, Kawagoe 350-8550, Japan

**Keywords:** national database, diagnosis, prescribed medication, cardiometabolic diseases, health checkups

## Abstract

The Japanese National Database (NDB), a useful data source for epidemiological studies, contains information on health checkups, disease diagnoses, and medications, which can be used when investigating common cardiometabolic diseases. However, before the initiation of an integrated analysis, we need to combine several pieces of information prepared separately into an all-in-one dataset (AIOD) and confirm the validation of the dataset for the study. In this study, we aimed to confirm the degree of agreement in data entries between diagnoses and prescribed medications and self-reported pharmacotherapy for common cardiometabolic diseases in newly assembled AIODs. The present study included 10,183,619 people who underwent health checkups from April 2018 to March 2019. Over 95% of patients prescribed antihypertensive and antidiabetic medications were diagnosed with each disease. For dyslipidemia, over 95% of patients prescribed medications were diagnosed with at least one of the following: dyslipidemia, hypercholesterolemia, or hyperlipidemia. Similarly, over 95% of patients prescribed medications for hyperuricemia were diagnosed with either hyperuricemia or gout. Additionally, over 90% of patients with self-reported medications for hypertension, diabetes, and dyslipidemia were diagnosed with each disease, although the proportions differed among age groups. Our study demonstrated high levels of agreement between diagnoses and prescribed medications for common cardiometabolic diseases and self-reported pharmacotherapy in our AIOD.

## 1. Introduction

In the last few decades, vast amounts of healthcare data have been stored and managed in digital electronic systems, along with the progression of database systems using upgraded computers and IT systems, which now allow us to encounter so-called healthcare big data [1,2,3].

The Japan National Database of Health Insurance Claims and Specific Health Checkups (NDB), provided by the Japanese Ministry of Health, Labour and Welfare (MHLW), contains data on all disease diagnoses and prescribed medications (administered medicinal substances) for the whole Japanese population, in addition to annual health checkup information [4]. The NDB is an important data source for epidemiological studies that aim to provide novel insights into the incidence of diseases, pathophysiology, and the underlying mechanisms for specific target diseases.

At the present time, the NDB is offered to investigators as separate datasets in the form of comma-separated value (CSV) files (datasets of health checkups, disease diagnoses, and prescribed medications) by the MHLW. Therefore, before the initiation of an integrated analysis, we need to assemble these datasets into an all-in-one dataset (AIOD) that includes disease diagnoses, prescribed medications, clinical features, and biochemical results (see graphical abstract).

Medication is typically prescribed post-diagnosis. Ideally, all patients prescribed a drug for a disease have a confirmed diagnosis, amounting to 100%. Nevertheless, this has not been confirmed yet, particularly for the newly prepared AIOD. Furthermore, the degree of reliability for self-reported medications on questionnaires administered during health checkups has also been poorly understood.

Therefore, in this study, we aimed to investigate the agreement in data entries between diagnoses and prescribed medications and self-reported medications in the AIOD, an assembled dataset obtained from several separate datasets from the NDB. Health checkups, whose data have been stored in the NDB, have been launched primally for the improvement of metabolic syndrome [5]. For several decades, hypertension, diabetes, and dyslipidemia have been major common cardiometabolic diseases that lead to fatal atherosclerosis and heart, brain, and vascular damage worldwide [6,7,8,9]; however, their underlying mechanisms and the associations between medications, clinical parameters, and lifestyles have not been fully revealed. Therefore, we investigated diagnoses and prescribed medications primarily for these three diseases in this study. This study did not investigate the appropriateness of prescribing for these common cardiometabolic diseases.

## 2. Materials and Methods

### 2.1. Study Design and Participants

The present study was a composite multidisciplinary study involving the secondary use of annual health checkup data in Japan as a part of the National Database Study in the Kanto 7 Prefectures (Tokyo, Kanagawa, Saitama, Chiba, Ibaraki, Gunma, and Tochigi) study (the NDB-K7Ps Study), which were collected to investigate clinical factors primarily associated with cardiometabolic diseases. Details of the study concept and design have been described elsewhere [10]. After a rigorous review of our research project by the MHLW, our protocol was accepted in December 2020 (No. 1320). We received digitally recorded anonymous data from the MHLW in July 2022.

In Japan, electronic submission of all insurance claims data from medical institutions has been mandatory according to the MHLW since 2011, with nearly complete penetration in 2015 (Figure S1 [11]). Therefore, the present study included nearly all claims data from 10,183,619 non-hospitalized individuals who were living in the above seven prefectures of Kanto and underwent specific health checkups from April 2018 to March 2019, which are mandatory for people aged 40–74 years in Japan [5]. During data processing to link the health checkup data with datasets comprising disease diagnoses and prescribed medications, we used special IDs prepared by the MHLW, which are unique to each person and described as hashed 64 alphanumeric codes based on sex, birth date, and insurance identification number. Data for diseases coded as “suspected” or “withdrawn” were excluded in this study. Data assembly via common ID and processing were performed using Excel CSV datasets offered by the MHLW and SAS datasheets that imported Excel data. Finally, we obtained an AIOD that included all relevant factors initially located in separate datasets.

### 2.2. Disease Diagnosis, Prescribed Medications, and Self-Reported Medication

In this study, we identified each disease using Japan’s disease codes, which are used for insurance claims in Japan [12] and correspond to a more detailed disease classification than the International Classification of Diseases, 10th revision (ICD-10) codes [13]. For example, type 2 diabetic nephropathy and renal failure were separately coded as “8830042” and “8845088”, respectively, in Japan’s disease codes, although they are coded as the same code (“E112”) in ICD-10. The disease codes in insurance claims were rigorously checked by medical clerks in each hospital and clinic. We defined patients with hypertension as those who were diagnosed with at least hypertension or essential hypertension [14]. Similarly, we defined patients with diabetes as those who were diagnosed with at least one of the 259 codes for diabetes (Table S1).

To investigate the proportion of diagnosed patients who were prescribed medications, we selected the following 19 medications: 8 antihypertensive medications (Trade names: Perdipine, Valsartan Tablets, RENIVACE Tablets, NU-LOTAN Tablets, Mikelan LA capsules, CALSLOT TABLETS, Selara Tablets, and PREMINENT Tablets) for hypertension, 3 antidiabetic medications (METGLUCO Tablets, TENELIA TABLETS, and Suglat Tablets) for diabetes, 3 medications for dyslipidemia (LIVALO OD TABLETS, BEZATOL SR Table and Zetia Tablets) for dyslipidemia, 2 medications for hyperuricemia and gout (Zyloric Tablets and Feburic Tablets), 2 vitamin K preparations (Glakay capsules and Kaytwo Capsules, Syrup and Injection), and 1 vitamin E preparation (Juvela Tablets, Capsules and Powder). Information about selected medications, namely, the trade name, nonproprietary name (Japanese Accepted Names for Pharmaceuticals [15]), therapeutic category, and clinical indications, was collected from the package inserts or the search system of the Prescription Medications in Pharmaceuticals and Medical Devices Agency [16].

Patients with self-reported medications for hypertension, diabetes, and dyslipidemia were defined as those who reported taking the following medicines on the questionnaire during health checkups: medications to reduce blood pressure, insulin injections or medications to reduce blood glucose, and medications to reduce cholesterol levels. The missing data about self-reported medications, which are considered data missing at random, were excluded from analysis.

### 2.3. Statistical Analysis

Accuracy was calculated by summing up all cases of agreement (number of individuals that have a diagnosis and medication, plus the number of individuals that have neither diagnosis nor medication) and dividing by the total number of individuals [17]. An individual’s response to the questionnaire depends on several factors. As sex and age are important influencing factors for cardiometabolic diseases [18,19,20,21], the proportion of patients who were diagnosed with hypertension, diabetes, and dyslipidemia among those who reported receiving pharmacotherapy on the questionnaire were compared between the four age groups (40s, 50s, 60s, and 70s) using the χ^2^ test with Bonferroni correction. Statistical analysis was performed using SAS-Enterprise Guide (SAS-EG 7.1) in SAS, version 9.4 (SAS Institute, Cary, NC, USA). Values of *p* < 0.05 were considered statistically significant. When differences in the proportion of patients between two selected groups among four age groups were evaluated by the χ^2^ test, values of *p* < 0.007 were considered to represent statistical significance on the basis of the Bonferroni test.

## 3. Results

### 3.1. Proportion of Diagnosed Patients among Those Who Were Prescribed Medications

Table 1 shows the proportion of diagnosed patients who were prescribed medications. The number of patients who were prescribed at least one of the eight antihypertensive medications was 51,598, while 1,982,782 reported receiving antihypertensive medication, 2,369,621 were diagnosed with hypertension, and 45,288 had overlaps among all three variables. Similarly, the number of patients who were prescribed at least one of the three antidiabetic medications was 114,597, while 515,636 reported taking antidiabetic medication, 1,293,487 were diagnosed with diabetes, and 96,900 had overlaps among all three variables. The number of patients who were prescribed at least one of the eight medications for dyslipidemia was 117,930, while 1,341,702 reported taking medication for dyslipidemia, 2,335,645 were diagnosed with dyslipidemia, and 93,724 had overlaps among all three variables.

Over 95% of patients prescribed antihypertensive medications were diagnosed with hypertension. Noteworthily, over 99% of patients prescribed antidiabetic medications were diagnosed with diabetes. In contrast, a low percentage (approximately 28%) of patients prescribed medication for dyslipidemia were diagnosed with dyslipidemia, with 24–65% for hypercholesterolemia (including familial hypercholesterolemia) and 40–71% for hyperlipidemia. In contrast, over 97% of patients prescribed medications for dyslipidemia were diagnosed when target diseases were replaced with at least one of those three diseases (dyslipidemia, hypercholesterolemia, or hyperlipidemia). Similarly, over 98% of patients prescribed medications for hyperuricemia were diagnosed with either hyperuricemia or gout. The accuracy was 77.2% in hypertension, 88.4% in diabetes, 78.2% in dyslipidemia, and 95.3% in hyperuricemia and gout.

Of the patients taking Glakay capsules (vitamin K_2_ preparation), 92.1% were diagnosed with osteoporosis, and 1.9% were diagnosed with vitamin K deficiency. Similarly, in patients treated with Juvela (vitamin E preparation), 2.9% were diagnosed with vitamin E deficiency.

### 3.2. Proportion of Diagnosed Patients among Those Who Reported Receiving Pharmacotherapy on the Questionnaire

Table 2 shows the proportion of patients who reported receiving pharmacotherapy for hypertension, diabetes, and dyslipidemia on the questionnaire during health checkups and those who were diagnosed with each disease. Overall, in all three diseases, the proportion of patients who reported receiving pharmacotherapy and who were diagnosed with each disease was over 90%. The accuracy was 93.3% for hypertension, 91.7% for diabetes, and 88.1% for dyslipidemia.

Like patients who were prescribed anti-dyslipidemia medications (Table 1), the proportion of patients who reported receiving pharmacotherapy for dyslipidemia, hypercholesterolemia, or hyperlipidemia was low (24–49%), whereas that of patients who were diagnosed with at least one of them was higher (91.8% in total).

Additionally, the proportion of diagnosed male patients who reported receiving pharmacotherapy (89.5–92.8%) was significantly lower than that of diagnosed female patients (94.1–94.7%) (χ^2^ test with Bonferroni correction for multiple comparisons, *p* < 0.007). Moreover, the proportion of diagnosed patients in their 40s who reported receiving pharmacotherapy (87.7–89.9%) was significantly lower than that of diagnosed patients in their 70s (94.4–94.9%) (χ^2^ test with Bonferroni correction for multiple comparisons, *p* < 0.007). In addition, there were more missing data for the questions about self-reported medications among people in their 40s than those in their 70s (Table S2).

## 4. Discussion

Although the NDB has covered over 95% of medical and dispensing claims for the Japanese population during the past decade (Figure S1 [11]), with the coverage reaching almost 100% in 2018 [22], little is known about the degree of agreement in data entries in an AIOD, an assembled NDB, which we used in [23] and will continue to use. Our study demonstrated a high level of agreement between diagnoses and prescribed medications for hypertension, diabetes, dyslipidemia (including hypercholesterolemia and hyperlipidemia), and hyperuricemia (including gout) in our assembled NDB datasheet. This suggests that the use of prescribed medications is reasonable as one of the confirmation methods for the diagnosis for further study using the NDB. Although “hyperlipidemia” was renamed “dyslipidemia” in 2007 in Japan [24], the previous term was still used for diagnosis in 2018. The prescription of the vitamin K preparation Glakay capsules was not in good agreement with the diagnosis of vitamin K deficiency, whereas it was in relatively high agreement with the diagnosis of osteoporosis, in accordance with clinical indications for Glakay [16]. Similarly, the vitamin E preparation Juvela is also used to treat arteriosclerosis and diabetic retinopathy. These results suggest that widely used medications, such as vitamin preparations, are not suitable for use as a means to validate a disease diagnosis, and that diseases without disease-specific medications, such as vitamin deficiency, are difficult to define using information on the prescribed medication.

Meanwhile, the proportion of patients having a diagnosed disease was not 100% among those who were administered medications, although it was over 95%. Although there was some time lag between the time points of the diagnosis entry and its revision by an attending physician and the actual administration of a drug, which might contribute to a few reductions from 100% (approximately 5%), it is unknown why such reductions were observed in our AIOD.

As one of the strong points for the NDB, it has detailed information available about medications, namely, the dosage, form, method of administration (powder, tablet, capsules, or injection), trade name, regardless of the original or generic product, and the pharmaceutical company that manufactures the drug. This information can reveal the frequency of prescribed medications, which might be useful in pharmacoeconomics as well as pharmacoepidemiology.

In this study, self-reported medications for hypertension, diabetes, and dyslipidemia on the health checkup questionnaire corresponded well with diagnoses, showing the high accuracy of self-reported medications. Although female individuals answered the questions in higher concordance with clinical diagnostic information than males, both proportions were almost over 90%. Interestingly, our results showed that individuals in their 70s answered the questions with the highest concordance with clinical diagnostic information (almost 95%) among the four age groups, whereas those in their 40s answered with the lowest (almost 90%). There were more missing data in the questions about self-reported medications among people in their 40s than those in their 70s (Table S2). Moreover, the proportion of individuals who underwent health guidance by medical staff, which is recommended for individuals at risk of cardiometabolic disease, was higher among people in their 70s than among those in their 40s [25]. Taken together, the results suggest that individuals in their 70s might be more conscious about promoting their own health and might complete the questionnaire more accurately than people in their 40s. Future studies targeting individuals in their 40s to investigate inaccuracies in self-reported medications are warranted.

Misclassification of the disease diagnosis is an important problem in claims databases [26,27,28,29]. Because matching between the NDB and other data sources is unfeasible in Japan, at least at the present time, several studies have been conducted to validate disease diagnoses using claims data from a single or several hospitals in which specialists in each disease confirm the condition by reviewing past medical records in detail. For example, high positive predictive values (almost 90%) were obtained in several studies by confirming both the diagnosis and treatment using disease-specific medications for type 1 diabetes, age-related macular degeneration, and medication-related osteonecrosis of the jaw [13,30,31]. Our study showed high positive predictive values (patients with diagnoses/patients with prescriptions (%): over 95%), which may support that our method is reasonably acceptable. Moreover, the diagnosis procedure combination case mix scheme (DPC) claims information, which is included in the NDB, may be useful for defining a disease without disease-specific medication, such as acute myocardial infarction [12], suggesting that further research involving DPC databases is needed.

Of note, ICD-10 codes were not always an appropriate method to identify a patient’s disease; for instance, viral, steroid, hepatic, secondary, drug-induced, and pancreatic diabetes were all categorized using the same code in ICD-10 (Table S1). Japan’s disease codes provide greater detail regarding the condition of a disease using a 7-digit code in comparison with ICD-10 codes, which is a strength of our study. However, misclassification is a serious problem, if any, in claims databases. Additionally, Japan’s disease codes include several unclear disease names, for instance, a diagnosis coded only as “diabetes mellitus” but without other information, which does not allow us to investigate the disease in detail.

The NDB, which comprises complete big data, is useful for investigating orphan diseases in which conditions, etiologies, and even epidemiological characteristics such as the prevalence rate are poorly understood because of inadequate numbers of observations and corresponding cases [32]. Future multidisciplinary analysis using the NDB, particularly the AIOD, will be helpful in obtaining a wide range of novel findings, confirming previous indefinite findings, and providing new perspectives for health promotion and disease prevention. To this end, it is crucial to assemble several NDB datasets and combine them into one AIOD through common IDs.

Several limitations should be mentioned in our study. First, it is impossible to confirm whether the actual diagnoses in the NDB were correct because linking the NDB with other databases, such as medical records at the individual level, is currently unavailable in Japan. Second, when the target disease has various pharmacotherapies and no disease-specific medications and, in particular, when pharmacotherapy is not essential for the target disease, our method is limited in use. Thirdly, there may be misreporting of self-medications due to recall bias, social desirability bias, and bias due to the level of health education/awareness in self-reported data. Finally, we had no available information about the duration of administration and the patient’s adherence to the medication, which can modify the effects of medications. Therefore, additional studies are needed to address these limitations.

## 5. Conclusions

Our results demonstrated a high level of agreement between diagnoses and prescribed medications for hypertension, diabetes, and dyslipidemia, common cardiometabolic diseases, and self-reported pharmacotherapy for these diseases, which suggests that the newly prepared AIOD is a reasonable and useful data source for epidemiological studies that will explore the association between diagnosed disease, prescribed medications, and clinical parameters, especially in terms of cardiometabolic diseases.

## Figures and Tables

**Table 1:** Proportion of diagnosed patients among those who were prescribed a specific medication.

Names of Diagnoses	Corresponding ICD-10 Code	Medications					Patients with Diagnosis/ Patients with Prescription, N (%)
		Trade Name			Nonproprietary Name ^1^	Therapeutic Category	
Hypertension ^2^	I10	Perdipine (Powder 10%, Tablets 10 mg/20 mg, LA Capsules 20 mg/40 mg, Injection 2 mg/5 mg/25 mg)			Nicardipine hydrochloride	Dihydropyridine calcium channel blocker	731/748 (97.7)
		Valsartan Tablets 20 mg (generic) ^3^			Valsartan	Angiotensin II receptor antagonist	7332/7423 (98.8)
		RENIVACE Tablets 2.5 mg/5 mg/10 mg			Enalapril Maleate	Angiotensin-converting enzyme inhibitor	5506/5796 (95.0)
		NU-LOTAN Tablets 25 mg/50 mg/100 mg			Losartan Potassium	Angiotensin II receptor antagonist	11,189/11,346 (98.6)
		Mikelan LA capsules 15 mg			Carteolol Hydrochloride	Beta-Adrenergic receptor antagonist (Beta blocker)	726/737 (98.5)
		CALSLOT TABLETS 5 mg/10 mg/20 mg			Manidipine Hydrochloride	Dihydropyridine calcium channel blocker	1308/1328 (98.5)
		Selara Tablets 25 mg/50 mg/100 mg			Eplerenone	Mineralocorticoid receptor antagonist	19,099/19,425 (98.3)
		PREMINENT Tablets LD/HD			Losartan Potassium/ Hydrochlorothiazide	Angiotensin II receptor blockers (ARBs) and diuretics	5509/5589 (98.6)
		Patients who were prescribed at least 1 of the above 8 antihypertensive medications					
					Medication		
		*n* (% column)			yes	no	
		Diagnosis		yes	50,620 (98.1)	2,319,001 (22.9)	
				no	978 (1.9)	7,813,020 (77.1)	Accuracy (%) * = 77.2%
Diabetes (except for gestational diabetes) ^4^	E10~14, E888	METGLUCO Tablets 250 mg			Metformin Hydrochloride	Biguanide antidiabetic	80,921/81,290 (99.5)
		TENELIA TABLETS 20 mg			Teneligliptin Hydrobromide Hydrate	DPP-4 inhibitor	34,691/34,849 (99.5)
		Suglat Tablets 25 mg			Ipragliflozin L-Proline	SGLT2 inhibitor	4936/4964 (99.4)
		Patients who were prescribed at least 1 of the above 3 antidiabetic medications					
					Medication		
		*n* (% column)			yes	no	
		Diagnosis		yes	114,067 (99.5)	1,179,420 (11.7)	
				no	530 (0.5)	8,889,602 (88.3)	Accuracy (%) * = 88.4%
Dyslipidemia	E785	LIVALO OD TABLETS 2 mg			Pitavastatin Calcium Hydrate	HMG-CoA reductase inhibitor	2158/7767 (27.8)
		BEZATOL SR Tab. 200 mg			Bezafibrate	Fibrate (PPAR alpha agonist)	2987/10,768 (27.7)
		Zetia Tablets 10 mg			Ezetimibe	Intestinal cholesterol transporter inhibitor	28,702/101,270 (28.3)
Hypercholesterolemia (including familial hypercholesterolemia)	E780	LIVALO OD TABLETS 2 mg			Pitavastatin Calcium Hydrate	HMG-CoA reductase inhibitor	5144/7767 (64.6)
		BEZATOL SR Tab. 200 mg			Bezafibrate	Fibrate (PPAR alpha agonist)	2690/10,768 (24.3)
		Zetia Tablets 10 mg			Ezetimibe	Intestinal cholesterol transporter inhibitor	64,524/101,270 (61.3)
Hyperlipidemia	E784, E785	LIVALO OD TABLETS 2 mg			Pitavastatin Calcium Hydrate	HMG-CoA reductase inhibitor	3073/7767 (39.6)
		BEZATOL SR Tab. 200 mg			Bezafibrate	Fibrate (PPAR alpha agonist)	7637/10,768 (70.9)
		Zetia Tablets 10 mg			Ezetimibe	Intestinal cholesterol transporter inhibitor	45,016/101,270 (44.5)
Dyslipidemia, hypercholesterolemia (including familial hypercholesterolemia), or hyperlipidemia	E780, E784, E785	LIVALO OD TABLETS 2 mg			Pitavastatin Calcium Hydrate	HMG-CoA reductase inhibitor	7685/7767 (98.9)
		BEZATOL SR Tab. 200 mg			Bezafibrate	Fibrate (PPAR alpha agonist)	10,415/10,768 (96.7)
		Zetia Tablets 10 mg			Ezetimibe	Intestinal cholesterol transporter inhibitor	99,462/101,270 (98.2)
		Patients who were prescribed at least 1 of the above 3 medications for dyslipidemia					
					Medication		
		*n* (% column)			yes	no	
		Diagnosis	yes		115,724 (98.1)	2,219,921 (22.1)	
			no		2206 (1.9)	7,845,768 (78.0)	Accuracy (%) * = 78.2%
Hyperuricemia	E790	Zyloric Tablets 50 mg/100 mg			Allopurinol	Uric acid biosynthesis inhibitor	13,788/17,407 (79.2)
		Feburic Tablets 10 mg/20 mg/40 mg			Febuxostat	Uric acid biosynthesis inhibitor	216,363/260,753 (83.0)
Gout (including gouty attack)	M1009	Zyloric Tablets 50 mg/100 mg			Allopurinol	Uric acid biosynthesis inhibitor	5909/17,407 (33.9)
		Feburic Tablets 10 mg/20 mg/40 mg			Febuxostat	Uric acid biosynthesis inhibitor	80,855/260,753 (31.0)
Hyperuricemia or gout	E790, M1009	Zyloric Tablets 50 mg/100 mg			Allopurinol	Uric acid biosynthesis inhibitor	17,042/17,407 (97.9)
		Feburic Tablets 10 mg/20 mg/40 mg			Febuxostat	Uric acid biosynthesis inhibitor	257,621/260,753 (98.8)
		Patients who were prescribed at 1 least the above 2 medications for hyperuricemia and gout					
					Medication		
		*n* (% column)			yes	no	
		Diagnosis	yes		273,685 (98.7)	472,650 (4.8)	
			no		3488 (1.3)	9,433,796 (95.2)	Accuracy (%) * = 95.3%
Vitamin K deficiency ^5^	E561, D684	Glakay capsules 15 mg			Menatetrenone	Vitamin K_2_ preparations/ Osteoporosis agent	39/2084 (1.9)
		Kaytwo (Capsules 5 mg, Syrup 0.2%, Injection 10 mg)				Vitamin K_2_ preparations	25/41 (61.0)
Osteoporosis	M8199	Glakay capsules 15 mg			Menatetrenone	Vitamin K_2_ preparations/ Osteoporosis agent	1919/2084 (92.1)
		Kaytwo (Capsules 5 mg, Syrup 0.2%, Injection10 mg)				Vitamin K_2_ preparations	— ^7^ (<20)
Vitamin E deficiency	E560	Juvela (Tablets 50 mg, Capsules 100 mg/200 mg, Powder 20%/40%)			Tocopherol acetate	Vitamin E preparations	1090/38,085 (2.9)
Arteriosclerosis	I709						1361/38,085 (3.6)
Diabetic retinopathy ^6^	E103, E113, E143						930/38,085 (2.4)

* Accuracy was calculated by summing up all cases of agreement (number of individuals that have a diagnosis and medication, plus the number of individuals that have neither diagnosis nor medication) and dividing by the total number of individuals. ^1^ Nonproprietary name according to Japanese Accepted Names for Pharmaceuticals [15]. ^2^ Hypertension included hypertension and essential hypertension. ^3^ Generic drugs made by 37 pharmaceutical companies. ^4^ Diabetes included the following 259 diagnoses, except for gestational diabetes: 60 type 1 diabetes mellitus, 61 type 2 diabetes mellitus, 10 viral diabetes mellitus, 10 steroid diabetes mellitus, 10 mitochondrial diabetes mellitus, 10 slowly progressive type 1 diabetes mellitus, 10 hepatic diabetes mellitus, 3 proliferative diabetic retinopathy, 48 diabetes mellitus, 10 secondary diabetes mellitus, 10 drug-induced diabetes, 10 pancreatic diabetes mellitus, 1 insulin-resistant diabetes mellitus, 1 stable diabetes mellitus, 1 brittle diabetes mellitus, 1 malnutrition-related diabetes mellitus, 1 fulminant type 1 diabetes mellitus, 1 juvenile type 2 diabetes, and 1 bronchogenic diabetes mellitus. ^5^ Vitamin K deficiency included deficiency of coagulation factor owing to vitamin K deficiency. ^6^ Diabetic retinopathy included the following nine diagnoses: diabetic retinopathy, type 1 diabetic central retinopathy, type 1 diabetic retinopathy, type 2 diabetic central retinopathy, type 2 diabetic retinopathy, proliferative diabetic retinopathy, proliferative diabetic retinopathy/type 1 diabetes, proliferative diabetic retinopathy/type 2 diabetes, and diabetic central retinopathy. ^7^ The detailed number is not expressed because of the small number of participants (<10), which could affect confidentiality. ICD-10, International Classification of Diseases, 10th revision.

**Table 2:** Proportion of diagnosed patients among those who reported receiving pharmacotherapy on the health checkup questionnaire.

Questionnaire of Specific Health Checkups	Names of Diagnoses	Corresponding ICD-10 Code	Patients with Diagnosis/Patients Reported Receiving Pharmacotherapy, N (%) ^†^					
			Sex		Age Groups (Years Old)			
Are You Taking Following Medicines at Present?			Men (M)	Women(W)	40–49	50–59	60–69	70–74
Q1. Medications to reduce blood pressure	Hypertension ^1^	I10	1,137,463/1,245,659 (91.3) *	697,913/737,123 (94.7) *	187,774/208,968 (89.9) *	474,685/523,370 (90.7)	1,172,917/1,250,444 (93.8)	483,587/509,394 (94.9) *
					Self-reported medication			
			*n* (% column)		yes	no		
			Diagnosis	yes	1,835,376 (92.6)	533,544 (6.5)		
				no	147,406 (7.4)	7,662,184 (93.5)	Accuracy (%) ^‡^ = 93.3	
Q2. Insulin injection or medications to reduce blood glucose	Diabetes ^2^	E10-14, E888	347,215/374,024 (92.8) *	133,202/141,612 (94.1) *	64,010/69,910 (91.6) *	133,200/144,054 (92.5)	283,207/301,672 (93.9)	109,367/115,696 (94.5) *
					Self-reported medication			
			*n* (% column)		yes	no		
			Diagnosis	yes	480,417 (93.2)	812,594 (8.4)		
				no	35,219 (6.8)	8,849,877 (91.6)	Accuracy (%) ^‡^ = 91.7	
Q3. Medications to reduce your cholesterol level	Dyslipidemia	E785	167,983/696,340 (24.1) *	150,248/645,362 (23.3) *	34,403/136,446 (25.2)	81,884/334,244 (24.5)	201,944/871,012 (23.2)	82,534/357,397 (23.1)
	Hypercholesterolemia ^3^	E780	323,350/696,340 (46.4) *	335,639/645,362 (52.0) *	64,300/136,446 (47.1)	162,899/334,244 (48.7)	442,912/871,012 (50.9)	183,445/357,397 (51.3)
	Hyperlipidemia	E784, E785	297,583/696,340 (42.7) *	271,638/645,362 (42.1) *	52,842/136,446 (38.7)	134,382/334,244 (40.2)	381,997/871,012 (43.9)	162,022/357,397 (45.3)
	Dyslipidemia, hypercholesterolemia, or hyperlipidemia	E780, E784, E785	623,231/696,340 (89.5) *	607,909/645,362 (94.2) *	119,709/136,446 (87.7) *	298,862/334,244 (89.4)	812,569/871,012 (93.3)	337,510/357,397 (94.4) *
					Self-reported medication			
			*n* (% column)		yes	no		
			Diagnosis	yes	1,231,140 (91.8)	1,103,615 (12.5)		
				no	110,562 (8.2)	7,732,880 (87.5)	Accuracy (%) ^‡^ = 88.1	

* Significant differences between sex or age groups were determined using χ^2^ test with Bonferroni correction for multiple comparisons (*p* < 0.007). ^†^ Proportions are calculated based on the available numbers for each question. ^‡^ Accuracy was calculated by summing up all cases of agreement (number of individuals that have a diagnosis and medication, plus the number of individuals that have neither diagnosis nor medication) and dividing by the total number of individuals. ^1^ Hypertension included hypertension and essential hypertension. ^2^ Diabetes included the following 259 diagnoses, except for gestational diabetes: 60 type 1 diabetes mellitus, 61 type 2 diabetes mellitus, 10 viral diabetes mellitus, 10 steroid diabetes mellitus, 10 mitochondrial diabetes mellitus, 10 slowly progressive type 1 diabetes mellitus, 10 hepatic diabetes mellitus, 3 proliferative diabetic retinopathy, 48 diabetes mellitus, 10 secondary diabetes mellitus, 10 drug-induced diabetes, 10 pancreatic diabetes mellitus, insulin-resistant diabetes mellitus, stable diabetes mellitus, brittle diabetes mellitus, malnutrition-related diabetes mellitus, fulminant type 1 diabetes mellitus, juvenile type 2 diabetes, and bronchogenic diabetes mellitus. ^3^ Hypercholesterolemia included familial hypercholesterolemia. ICD-10, International Classification of Diseases, 10th revision.

## Data Availability

Any inquiries regarding the availability of the data and the code to merge the datasets in this study should be directed to the corresponding author.

## Supplementary Materials

The following supporting information can be downloaded at https://www.mdpi.com/article/10.3390/epidemiologia4040034/s1: Figure S1: Trends in the penetration rate of electronically submitted health insurance claims data from medical institutions.; Table S1: Detailed description for the diagnosis of diabetes (translated from Japanese to English).; Table S2: Proportion of missing data in the questions about self-reported medication.
