# Supplementary material for: Agreement in All-in-One Dataset between Diagnosis and Prescribed Medication for Common Cardiometabolic Diseases in the NDB-K7Ps
Source: Epidemiologia (Basel). 2023 Oct 2;4(4):370–81. doi: 10.3390/epidemiologia4040034 (PMC10594522; doi:10.3390/epidemiologia4040034)
Supplement: Supplementary file 1 [file epidemiologia-04-00034-s001.zip › epidemiologia-2540846-supplementary.pdf]

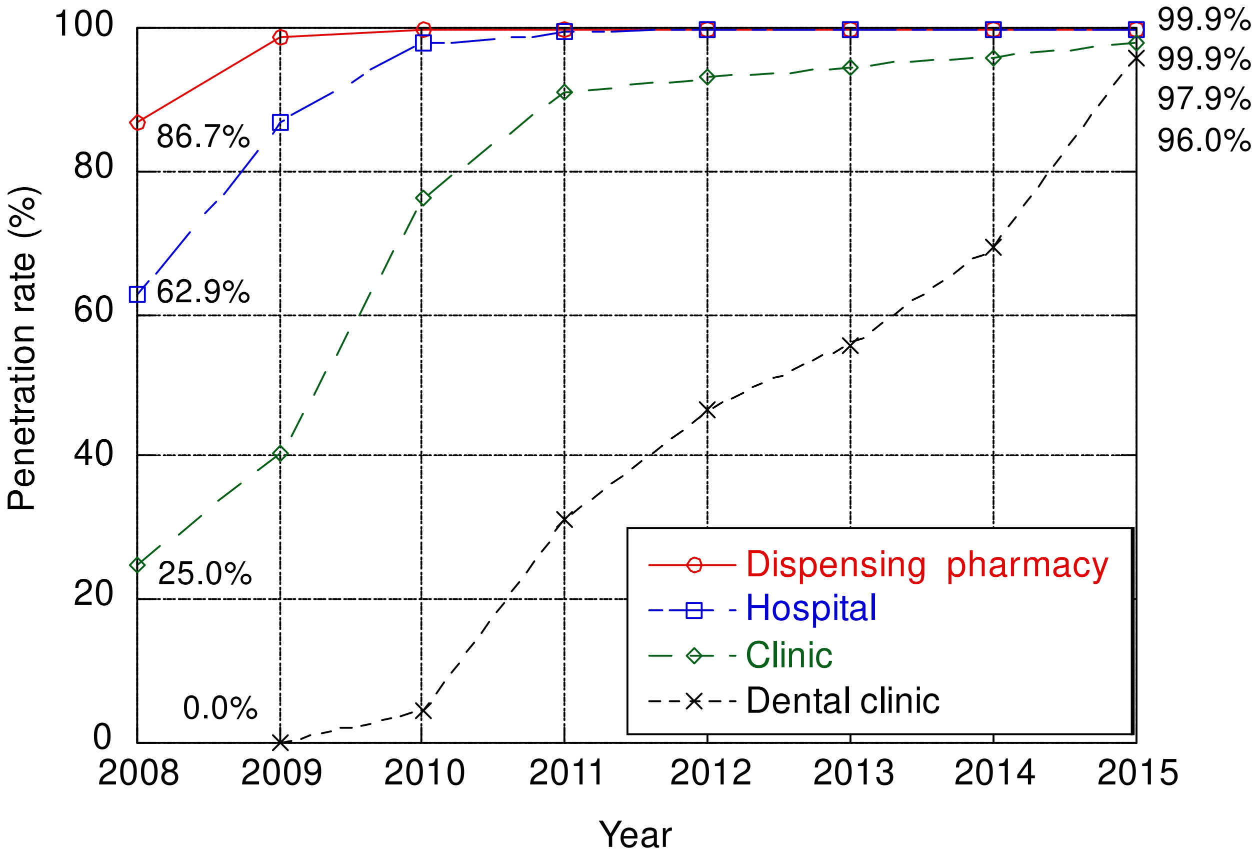

Fig S1. Trends in the penetration rate of electronically submitted health insurance claims data from medical institutions.

This figure was created by processing “Trends in the penetration rate of electronically submitted health insurance claims data from medical institutions (based on the number of receipts)” [11].

Table S1. Detailed description for the diagnosis of diabetes (translated from Japanese to English).

Subcategories (n of detail diagnosis name)	Detail name of diagnoses	Corresponding ICD-10 code
Type 1 diabetes mellitus (60)	Type 1 diabetes mellitus; Type 1 diabetes mellitus with ketoacidosis, with joint complications, with ocular complications, with coma complications, with neurological complications, with kidney complications, with multiple diabetic complications, with diabetic complications with peripheral circulatory complications and without diabetic complications;<br>Type 1 diabetic maculopathy, acidosis, acetonemia, ketoacidosis, ketosis, pruritus, neuropathy, gastroenteropathy, macular edema, gangrene, liver disorder, arthropathy, ocular muscle paralysis, amyotrophy, vasculopathy, hypercholesterolemia, hyperglycemia-hyperosmotic syndrome, osteopathy, diabetic coma, autonomic neuropathy, neurogenic bladder, neuralgia, glomerulosclerosis, nephropathy, nephropathy stage 1/ 2/ 3/ 3A/ 3B/ 4/ 5, renal failure, blistering, mental disorder, polyneuropathy, mononeuropathy, central retinopathy, ulcer, hypoglycemic coma, arteriosclerosis, arterial occlusive disease, iritis, cataract, skin disorder, edematous sclerosis, peripheral vasculopathy, peripheral vascular disease, peripheral neuropathy, and retinopathy	E10, E100-107, E109
Type 2 diabetes mellitus (61)	Type 2 diabetes mellitus; Type 2 diabetes mellitus with ketoacidosis, with joint complications, with ocular complications, with coma complications, with neurologic complications, with renal complications, with multiple diabetic complications, with diabetic complications, with peripheral circulatory complications and without diabetic complications;<br>Type 2 diabetic maculopathy, acidosis, acetonemia, ketoacidosis, ketosis, pruritus, neuropathy, myopathy, gastroenteropathy, macular edema, gangrene, liver disorder, arthropathy, ocular muscle paralysis, amyotrophy, vasculopathy, hypercholesterolemia,	E11, E110-117, E118

	hyperglycemia-hyperosmotic syndrome, osteopathy, coma, autonomic neuropathy, neurogenic bladder, neuralgia, nephrosclerosis, nephropathy, nephropathy stage 1/ 2/ 3/ 3A/ 3B/ 4/ 5, renal failure, blistering, mental disorder, polyneuropathy, mononeuropathy, central retinopathy, ulcer, hypoglycemic coma, arteriosclerosis, arterial occlusive disease, iritis, cataract, skin disorder, edematous sclerosis, peripheral vasculopathy, peripheral vascular disease, peripheral neuropathy and retinopathy	
Viral diabetes mellitus (10)	Viral diabetes mellitus; Viral diabetes mellitus with ketoacidosis, with ocular complications, with coma, with neurologic complications, with renal complications, with multiple diabetic complications, with diabetic complications, with peripheral circulatory complications and without diabetic complications	E13, E130-137, E139
Steroid diabetes mellitus (10)	Steroid diabetes mellitus; Steroid diabetes mellitus with ketoacidosis, with ocular complications, with coma, with neurologic complications, with renal complications, with multiple diabetic complications, with diabetic complications, with peripheral circulatory complications and without diabetic complications	E13, E130-137, E139
Mitochondrial diabetes mellitus (10)	Mitochondrial diabetes mellitus; Mitochondrial diabetes mellitus with ketoacidosis complications, with ocular complications, with coma complications, with neurological complications, with renal complications, with multiple diabetic complications with diabetic complications, with peripheral circulatory complications and without diabetic complications	E888
Slowly progressive type 1 diabetes mellitus (10)	Slowly progressive type 1 diabetes mellitus; Slowly progressive type 1 diabetes mellitus with ketoacidosis, with joint complications, with ocular complications, with coma complications, with neurologic complications, with renal complications, with multiple diabetic complications, with peripheral circulatory complications and without diabetic complications	E10, E100-107, E109
Hepatic diabetes mellitus	Hepatic diabetes mellitus; Hepatic diabetes mellitus with ketoacidosis, with ocular	E13, E130-

(10)	complications, with coma, with neurological complication, with renal complications, with multiple diabetic complications, with diabetic complications, with peripheral circulatory complications and without diabetic complications	137, 139
Proliferative diabetic retinopathy (3)	Proliferative diabetic retinopathy; Proliferative diabetic retinopathy/ type 1 diabetes; Proliferative diabetic retinopathy/ type 2 diabetes	E103, E113, E143
Diabetes mellitus (48)	Diabetes mellitus; Diabetes without diabetic complications; Diabetic maculopathy, macular edema, complications, acidosis, acetonemia, ketoacidosis, ketosis, pruritus, neuropathy, gangrene, liver damage, arthropathy, ocular muscle paralysis, amyotrophy, vasculopathy, hypercholesterolemia, osteopathy, coma, autonomic neuropathy, neurogenic bladder, neuropathic pain, neuralgia, nephrosclerosis, nephropathy, kidney disease, renal failure, blistering, mental disorder, polyneuropathy, mononeuropathy, central retinopathy, ulcer, hypoglycemic coma, arteriosclerosis, arterial occlusive disease, iritis, cataract, skin disorder, edematous sclerosis, peripheral vasculopathy, peripheral vascular disease, peripheral neuropathy, foot gangrene, foot ulcer, foot lesions, retinopathy	E14, E140-147, E149
Secondary diabetes mellitus (10)	Secondary diabetes mellitus; Secondary diabetes mellitus with ketoacidosis, with ocular complications, with coma, with neurological complications, with renal complications, with multiple diabetic complications, with diabetic complications, with peripheral circulatory complications and without diabetic complications	E13, E130-137, E139
Drug-induced diabetes (10)	Drug-induced diabetes; Drug-induced diabetes mellitus with ketoacidosis, with ocular complications, with coma, with neurological complications, with renal complications, with multiple diabetic complications, with diabetic complications, with peripheral circulatory complications and without diabetic complications	E13, E130-137, E139

Pancreatic diabetes mellitus (10)	Pancreatic diabetes mellitus; Pancreatic diabetes mellitus with ketoacidosis, with ocular complications, with coma, with neurological complications, with renal complications, with multiple diabetic complications, with diabetic complications, with peripheral circulatory complications and without diabetic complications	E13, E130-137, E139
Others (7)	Insulin-resistant diabetes mellitus	E11
	Stable diabetes mellitus	E11
	Brittle diabetes mellitus	E10
	Malnutrition-related diabetes mellitus	E12
	Fulminant type 1 diabetes mellitus	E101
	Juvenile type 2 diabetes	E11
	Bronchogenic diabetes mellitus	E831

ICD-10, International Classification of Diseases, 10th revision.

Table S2. Proportion of missing data in the questions about self-reported medication

	N (% of each group)				
Questionnaire of specific health checkups	Age groups (years old)				
Are you taking following medicines at present?	All	40-49	50-59	60-69	70-74
Q1. Medications to reduce blood pressure	2,634 (0.05)	2,414 (0.07)	1,616 (0.05)	859 (0.04)	220 (0.02)
Q2. Insulin injection or medications to reduce blood glucose	2,724 (0.06)	2,459 (0.07)	1,763 (0.06)	1,025 (0.04)	265 (0.02)
Q3. Medications to reduce your cholesterol level	2,698 (0.06)	2,447 (0.07)	1,755 (0.06)	969 (0.04)	251 (0.02)

All differences in proportion of missing data in the questions between people in their 40s and the other age groups were significant ($p < 0.007$, according to χ^2 test with Bonferroni correction for multiple comparisons).
